# ZSeeker: an optimized algorithm for Z-DNA detection in genomic sequences

**DOI:** 10.1093/bib/bbaf240

**Published:** 2025-05-30

**Authors:** Guliang Wang, Ioannis Mouratidis, Kimonas Provatas, Nikol Chantzi, Michail Patsakis, Ilias Georgakopoulos-Soares, Karen M Vasquez

**Affiliations:** Division of Pharmacology and Toxicology, Dell Pediatric Research Institute, College of Pharmacy, The University of Texas at Austin, 1400 Barbara Jordan Boulevard, Austin, TX 78723, United States; Institute for Personalized Medicine, Department of Biochemistry and Molecular Biology, The Pennsylvania State University College of Medicine, 500 University Drive, Hershey, PA 17033, United States; Huck Institutes of the Life Sciences, Pennsylvania State University, 201 Huck Life Sciences Building, University Park, PA 16802, United States; Huck Institutes of the Life Sciences, Pennsylvania State University, 201 Huck Life Sciences Building, University Park, PA 16802, United States; Institute for Personalized Medicine, Department of Biochemistry and Molecular Biology, The Pennsylvania State University College of Medicine, 500 University Drive, Hershey, PA 17033, United States; Huck Institutes of the Life Sciences, Pennsylvania State University, 201 Huck Life Sciences Building, University Park, PA 16802, United States; Huck Institutes of the Life Sciences, Pennsylvania State University, 201 Huck Life Sciences Building, University Park, PA 16802, United States; Institute for Personalized Medicine, Department of Biochemistry and Molecular Biology, The Pennsylvania State University College of Medicine, 500 University Drive, Hershey, PA 17033, United States; Huck Institutes of the Life Sciences, Pennsylvania State University, 201 Huck Life Sciences Building, University Park, PA 16802, United States; Division of Pharmacology and Toxicology, Dell Pediatric Research Institute, College of Pharmacy, The University of Texas at Austin, 1400 Barbara Jordan Boulevard, Austin, TX 78723, United States

**Keywords:** Z-DNA, algorithm design, search tool, web interface

## Abstract

Z-deoxyribonucleic acid (Z-DNA) is an alternative left-handed DNA structure with a zigzag-shaped backbone that differs from the right-handed canonical B-DNA helix. Z-DNA has been implicated in various biological processes, including transcription, replication, and DNA repair, and can induce genetic instability. Repetitive sequences of alternating purines and pyrimidines have the potential to adopt Z-DNA structures. ZSeeker is a novel computational tool developed for the accurate detection of potential Z-DNA-forming sequences in genomes, addressing key limitations of prior methods, such as computational inefficiency, difficult interpretability and usability, and lack of experimentally generated data. By introducing a novel methodology informed and validated by experimental data, ZSeeker enables the refined detection of potential Z-DNA-forming sequences. Built both as a standalone Python package and as an accessible web interface, ZSeeker allows users to input genomic sequences, adjust detection parameters, and view potential Z-DNA sequence distributions and Z-scores via downloadable visualizations. Our web platform provides a no-code solution for Z-DNA identification, with a focus on accessibility, user-friendliness, speed, and customizability. By providing efficient, high-throughput analysis, and enhanced detection accuracy, ZSeeker has the potential to support significant advancements in understanding the roles of Z-DNA in normal cellular functions, genetic instability, and its implications in human diseases.

## Introduction

Since the discovery of the canonical right-handed B-deoxyribonuceic acid (B-DNA) double helical structure, a number of alternative DNA conformations (i.e. non-B DNA) that differ from the canonical “Watson-Crick” B-form DNA structure [1] (e.g. Z-DNA, H-DNA, G4-DNA, and hairpin/cruciform DNA) have been characterized [2–4]. Z-DNA, a left-handed DNA structure [5], plays important roles in various genomic functions and has profound implications for transcription, replication, nucleosome positioning, recombination, and DNA damage and repair [6–9]. Z-DNA-forming sequences have been shown to stimulate genetic instability and are enriched at mutation hotspots in human cancer genomes, implicating them in the etiology of human diseases [10–15].

Alternating purine-pyrimidine sequences have the capacity to adopt Z-DNA structures, in which the purine residues are in the syn-conformation and pyrimidines in the anti-conformation. The sugar-phosphate backbone of the DNA helix is twisted into a left-handed zigzag-shape by the alternating syn- and anti- conformations [16]. However, it has been realized that not all alternating purine-pyrimidine dinucleotides have thermodynamic features that promote Z-DNA formation, with GC being the most favored, followed by GT/AC, and AT the least stable [17, 18].

Based on the unique sequence features required for Z-DNA formation, multiple sequence-based computer algorithms, evaluating sequence type and length were developed to search for potential Z-DNA-forming sequences in genomes [19, 20]. The energetic parameters for stabilizing all 16 possible dinucleotides in the Z-DNA conformation were measured [19] and this information was used to develop the well-recognized “Z-Hunt” program to predict potential Z-DNA-forming sequences in genomes [18].

The development of the Z-Hunt program (and later the updated Z-Hunt II program) was a major step forward for computer-aided DNA structure prediction and was recognized as the most accepted model because it was based on the energetic requirements for Z-DNA formation estimated from experimental data. However, certain limitations of these foundational studies should be noted. For example, the energetic measurements of Z-DNA formation *in vitro* that were used for the Z-Hunt II algorithm design were achieved by two-dimensional agarose gel electrophoresis in Tris-borate-EDTA (TBE) buffer (or TBE buffer containing 1.3 μM chloroquine), which does not contain physiological Z-DNA-stabilizing divalent cations or B-DNA-favoring alkali cations. The algorithms predict the formation of Z-DNA by analyzing the transition of all 16 possible dinucleotides using the energetic measurements obtained above. In addition, these experiments were performed on plasmid DNA used for these early studies containing multiple non-B DNA-forming motifs in addition to Z-DNA that could compete with each other in the structure transition measurements, confounding the interpretation of the Z-DNA-forming potential. Further, the algorithm was developed based on knowledge and technology several decades ago, and thus has several limitations for the accurate prediction of Z-DNA-forming sequences. For example, the Z-Hunt II algorithm searches long sequences by subdividing the sequences into fixed search windows of 16–24 bps and evaluating the Z-DNA-forming potential in each window. Although the windows have overlap to reduce the possibility of dissecting Z-DNA-forming motifs between two adjacent windows, it natively has limitations in identifying long Z-DNA-forming sequences, particularly those that have sequence interruptions but still maintain Z-DNA-forming capabilities. Also, Z-Hunt II examines each window independently, calculating the energies for Z-DNA formation at the dinucleotide level and does not account for potential longer-range effects, such as DNA bending forces [21]. In addition, the energy requirements for forming a Z-DNA structure are more complicated than adding the energy for each dinucleotide. For example, the unpaired and extruded bases at the B–Z junctions allow the adjacent Z-DNA segment to base stack on the neighboring B-DNA segment, which requires more energy than maintaining the alternating purine-pyrimidine dinucleotide in the Z-DNA formation [22]. Therefore, the average free energies for each dinucleotide in a longer Z-DNA-forming motif may be less than the same dinucleotide in a short motif, which also must flip two B–Z junctions. However, when two Z-DNA motifs with scores X and Y are put together, the merged sequence received a combined score of X + Y in Z-Hunt II, even though the merging reduces to two out of four energy-consuming B–Z junctions.

In addition to Z-Hunt, several other programs are available. For example, the National Cancer Institute (NCI) non-B DB (https://nonb-abcc.ncifcrf.gov/apps/site/resources) Genomic Database Search Tool, which also uses Z-Hunt II principles for Z-DNA searching and scoring [20], but gives significantly different predictions to Z-Hunt II, as discussed later in our comparison section.

Recently, deep learning approaches were implemented to search for potential Z-DNA-forming sequences [23]. The accuracy of deep learning approaches depends on the availability of high-quality large datasets for training. The currently available datasets were based on the recognition of Z-DNA structures that were formed at the particular time of the experiment, including the single-stranded DNA sequencing datasets [24, 25] or the Permanganate/S1 nuclease footprinting dataset [26] that are used for training the DeepZ program [23]. While these are useful and informative datasets, they are more appropriate for demonstrating the formation of non-B DNA under certain conditions. In addition, the Z-DNA-binding proteins used in the ChIP-seq experiments to develop the programs, such as adenosine deaminase acting on RNA 1 (ADAR1) or the Z-DNA binding domain of human DNA-dependent activator of IFN-regulatory factors (DAI) (ZBP1) are not passive Z-DNA binding proteins, but can actively convert B-DNA into Z-DNA [24]. For example, ZBP1 was able to convert a 14-bp B-DNA duplex to Z-DNA, which was used for crystal structure analysis [27]. Therefore, Z-DNA structure datasets used for training the deep-learning programs have some limitations, which we also discuss in our comparison section. In summary, although multiple different approaches are available to identify potential Z-DNA-forming sequences, a more accurate, easy-to-use, and reliable tool to predict Z-DNA structure formation is warranted.

**Figure 1:**
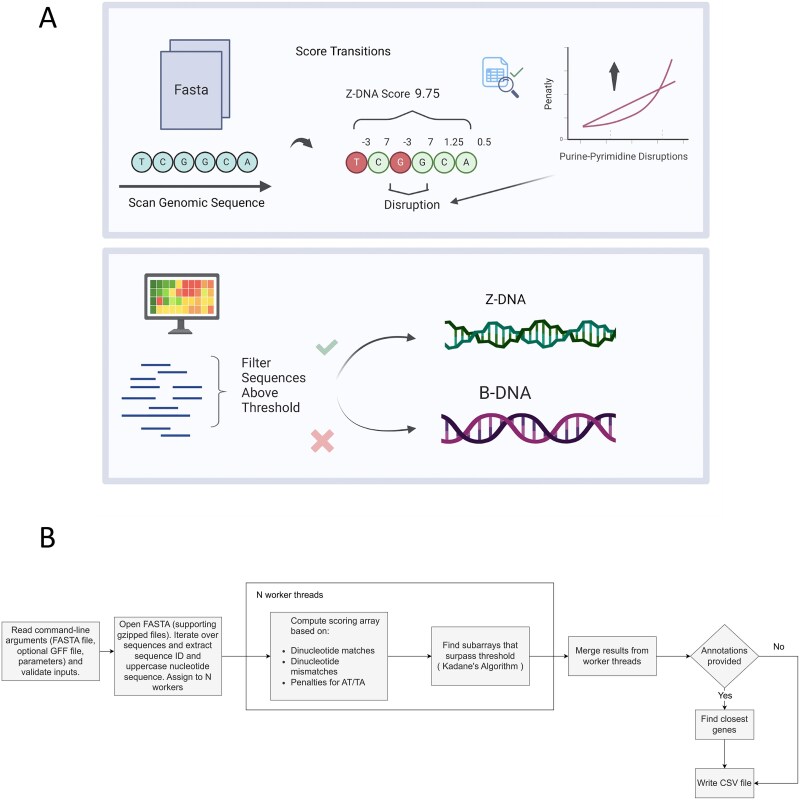
Schematic representation of B-DNA and Z-DNA conformations as well as the algorithmic design of ZSeeker as a flowchart. (A) The schematic illustrates the scoring algorithm and penalties that ZSeeker allows. (B) Flowchart of ZSeeker methods, parameters, and penalties.

Building on published experimental thermodynamic data generated previously, here we also included data from biochemical and genetic experiments of Z-DNA formation from different systems, along with advanced computational processing ability, to design a novel and optimized Z-DNA-finder program. We introduce “ZSeeker”, a Python package that enables rapid and accurate identification of potential Z-DNA-forming sequences. We also provide a web portal for analyzing sequences for Z-DNA structure-forming potential, visualizing, and downloading the outputs (Fig. 1). We compare the accuracy and efficiency of Z-DNA searching and prediction of ZSeeker with Z-Hunt (II), non-B DB, and Z-DNABERT and report that ZSeeker allows for a more accurate and flexible Z-DNA prediction and searching platform than the currently existing programs. Through ZSeeker, we aim to support the scientific community in advancing Z-DNA research and its applications.

## Materials and methods

### Linear sequence feature for Z-DNA structure prediction

The succession of bases within repetitive sequences dictates the potential interactions among different regions in a DNA molecule and determines its potential for the formation and stability of non-B DNA structures. An alternating purine-pyrimidine (e.g. GC or GT) repeat sequence has the propensity to adopt a Z-DNA structure, in which purine residues are in the syn-conformation and pyrimidines are in the anti-conformation. The alternating syn- and anti-formations twist the sugar-phosphate backbone into a left-handed zigzag-shape [16]. Under certain conditions such as base modifications or the presence of ions, Z-DNA structures can also form on regions that are not typical alternating purine-pyrimidine patterns, such as CCG/CGG or CGATCG repeats [28, 29]. Although the formation of Z-DNA is affected by many other factors such as salt, pH, negative supercoiling, and the presence of binding proteins and need to be taken into consideration, the linear sequence feature is the premise for the ability of a sequence to adopt secondary structural conformations, and provides the fundamental information for computing the scores for contributions and penalties for each transition between the adjacent nucleotides in Z-DNA formation.

### Intracellular processing of Z-DNA as an indicator for Z-deoxyribonucleic acid structure formation

Since the energetic parameters from biochemical experiments do not fully mimic the complex conditions *in vivo*, and detecting Z-DNA formation directly in living cells is challenging, we used Z-DNA-induced mutagenesis as an indirect indicator for Z-DNA formation *in viv*o. We found that a CG(5) repeat cloned into the *lacZ’* gene in a mutation reporter was ~30× more mutagenic than control B-DNA (puCON) in bacterial DH5alpha cells, and when the CG repeats were increased to 14 units CG(14), the mutation frequencies increased further in bacteria, mammalian cells, and in mice [11, 30]. S1 enzyme probing and chloroquine gel electrophoresis analyses also confirmed Z-DNA formation in the CG(14) repeat. Under the same conditions, a GT(14) repeat was not mutagenic (Fig. S1), and a GT20 repeat cloned in a *supF* mutation reporter only induced slightly higher mutation frequencies than the control B-DNA sequence in mammalian COS-7 cells. In addition, most of the mutants were small indels within the repeats (Fig. S2), likely due to slipped strand mispairing occurring on simple repeats [31], rather than the signature Z-DNA-induced large deletions resulting from DNA double-strand breaks (DSBs) as seen in mammalian cells [10, 11], suggesting a lack of stable Z-DNA formation *in vivo*. However, when the GT repeat reached a length of 30 (GT30), >50% of the mutants were large deletions, the signature Z-DNA-induced mutation in mammalian cells. A longer GT41 repeat induced similar levels of mutations in mammalian COS-7 cells as the CG14 sequence, and the majority of the mutants were large deletions, providing strong evidence for Z-DNA formation in mammalian cells [32]. In summary, our *in vivo* experiments suggested more substantial differences between the GC and GT repeats where GC sequences only required 4–5 repeats to form a Z-DNA structure (Fig. S1), while GT sequences required 20–30 repeats (we consider 25 repeats as a minimum length) to adopt a Z-DNA structure (Fig. S2), consistent with previous reports [33–35]. For AT dinucleotides, we found that a GC repeat containing two embedded AT repeats can still form Z-DNA [36]. However, it was found that an AT4 repeat, even when located between two CG6 repeats, adopted an unwound structure rather than a Z-DNA structure [37], and an AT14 repeat adopted a slippage/bubble structure instead of Z-DNA, resulting in small indels within the repeats rather than large deletions (Fig. S2). In addition to the scores of each transition, ZSeeker takes into account global sequence features that are not available in the previous models. For example, it identifies consecutive AT runs and scores each differently. The penalty score also changes after each additional one. It also considers and favors long runs of combined adjacent Z-motifs, so that only 2 B–Z junctions are needed, to avoid the high energy requirement for additional junctions if the interruptions were considered as B-DNA. Based on these historical and current experimental datasets, we designed our new ZSeeker prediction and searching program.

**Table 1:** Recommended parameters for Z-DNA searching

Parameter	Description	Value
Threshold	Scoring threshold for a sequence to be considered a potential Z-DNA-forming sequence and returned by the program	50
GC weight	Weight given to GC and CG transitions	7
AT weight	Weight given to AT and TA transitions	0.5
Consecutive AT scoring^a^	Penalty array for consecutive AT/TA repeats to account for hairpin structures. Provides the score adjustment for each AT/TA transition	0.5, 0.5, 0.5, 0, 0, −5, −100
AC weight	Weight given to AC and CA transitions	1.25
GT weight	Weight given to GT and TG transitions	1.25
Mismatch penalty starting value^b^	Penalty applied to the first non-purine/pyrimidine transition encountered	3
Mismatch penalty type	Method of scaling the penalty for contiguous non-purine/pyrimidine transitions	Linear or Exponential
Mismatch penalty linear delta^c^	Rate of increase of the penalty for each subsequent non-purine/pyrimidine transition (only applies if penalty type is linear)	3
GFF file	Optional GFF file for gene annotation. Only “gene” features are used.	None
Drop threshold	Drop threshold used within subarrays detection logic. Acts as earlier stopping threshold. Lower values result in smaller Z-DNA sequences and larger values result in fewer but larger Z-DNA sequences.	50
Total sequence scoring	If set, calculate the total score of all provided sequences, without looking for Z-DNA subsequences. Useful for researchers who have short sequences and want to estimate their Z-DNA potential.	False

Note: Non-B DNA formation is a dynamic process and is not decided solely by the DNA sequence. Cellular activities such as replication, transcription, and DNA repair can impact non-B DNA formation via changing DNA supercoiling levels, affecting DNA binding proteins, and modulating nucleosome/chromatin structures. The results from this algorithm only provide initial information on the potential of a given DNA sequence to form a Z-DNA structure.

^a^ **Consecutive AT scoring:** Because over 4 consecutive AT/TA repeats tend to form hairpin structures instead of Z-DNA [37], a penalty array is defined, which provides the score adjustment for the first and the subsequent AT/TA transitions. The last element will be applied to every subsequent AT/TA repeat.

^b^ **Mismatch penalty starting value:** Penalty applied to the first non purine/pyrimidine transition encountered: 3.

^c^ **Mismatch penalty linear delta:** Only applies if penalty type is set to linear. Determines the rate of increase of the penalty for every subsequent non purine/pyrimidine transition: 3.

### Algorithmic process for Z-DNA detection

The algorithm begins by reading nucleotide sequences from a FASTA file and computing a scoring array based on dinucleotide transitions. For each pair of consecutive nucleotides, the algorithm assigns specific weights to favorable transitions such as GC/CG, GT/TG, and AC/CA; in contrast, unfavorable transitions (including pairs like GG or CC) or other non-purine-pyrimidine transitions (termed mismatches) incur a penalty that increases in a linear (or optionally exponential) manner. When an AT or TA transition is encountered, the algorithm checks for consecutive occurrences and adds an additional penalty from a predefined penalty array, using the final penalty value when the count exceeds the array length to simulate the fact that more than four AT repeats are unlikely to form Z-DNA as described above. Maximum scoring subarrays that exceed a specified threshold are candidates for Z-DNA formation, and if multiple subarrays meet the threshold, a dynamic programming approach selects the longest subarray through Kadane’s algorithm, identifying maximum length potential Z-DNA-forming sequences. To improve performance, the computation is parallelized both when processing sequences per chromosome and when determining the maximum subarrays, as well as when finding the closest gene annotations if a General feature format (GFF) file is provided. In the annotation step, the GFF file is parsed and gene coordinates are converted from 1-based to 0-based intervals, and for each Z-DNA candidate, the nearest gene is assigned by rapidly searching through sorted gene coordinates while taking strand orientation into account for transcription start site (TSS) and transcription end site (TES) calculations. The parameters, such as the weights of contributions or penalties for each transition, and effects of the lengths of consecutive repeats were optimized based on the previously published biochemical energetic measures and from biological experiments. The default settings are described below, but are still open for fine adjustment for best performance under users’ specific situations.

### Recommended prediction parameters

This algorithm scores the potential Z-DNA-forming sequences primarily based on the transitions between the bps that support or prevent Z-DNA formation. Since a GC(4) repeat has 7 “G to C transitions”, and a GT(25) repeat has 49 “G to T transitions”, and both are minimum lengths for Z-DNA conformation, this algorithm gives each G to C or C to G transition a score of “7”, each “G to T” or “T to G” transition a score of “1.25”, and sets the threshold for Z-DNA formation at “50”. An “A to T” or “T to A” transition scores 0.5 (Table 1). However, a continuous AT or TA run over 4 repeat units is more likely to form hairpins or cruciform structures, and therefore has a high penalty score (Table 1). A sequence obtaining a score over the threshold will be considered as a candidate sequence. The algorithm is designed to identify the DNA subsequences that achieve the highest score and mark them as potential Z-DNA-forming sequences**.** The recommended parameters for Z-DNA searching are provided below in Table 1.

### Sensitivity analysis

A comprehensive sensitivity analysis was conducted to evaluate the performance of ZSeeker in predicting potential Z-DNA-forming sequences. The study employed a set of sequences derived from wet-lab experiments and Z-DNA related publications, with positive controls expected to form Z-DNA and negative controls designated by a “non” prefix in the FASTA file. The origin of each sequence can be found in Supplementary Table S1. Dinucleotide weights were systematically varied across defined ranges: GC weights were tested from 4 to 9 (with a default centered at 7); GT and AC weights were explored over the range of 0.5 to 2; and the AT weight was fixed at 0.5 due to its nonlinear relationship with consecutive AT transitions. All possible combinations of these parameters were assessed to determine the sensitivity and specificity of the tool, with predictions based on a threshold Z-DNA score (greater than 50 for forming and less than 50 for nonforming sequences). The outcomes of each parameter set were aggregated into a summary Comma-separated values (CSV) file, and the entire analysis can be reproduced by executing the provided Python script. The results of the sensitivity analysis can be found in Supplementary Table S2.

### Command-line interface (CLI) tool and package

ZSeeker is available both as a command-line interface tool and as a Python library, distributed freely as an open-source package on PyPI (https://pypi.org/project/ZSeeker/). Users can install ZSeeker with a single command and choose to use it through the command line or integrate it into their Python code. Both options allow users to adjust the tool’s performance by utilizing multiprocessing to evaluate input sequences more efficiently.

### ZSeeker web interface

The ZSeeker web interface provides a straightforward, accessible version of the ZSeeker command-line tool intended for small-scale analyses and visualizations (files up to 15 MB). It uses the Gin framework in Go for the backend and HTML, CSS, and JavaScript for the frontend. The main purpose of the web application is to make ZSeeker’s features easy to use. Users can submit jobs, inspect, download, and filter Z-DNA results directly through the interface. It also includes visualizations to help users identify and evaluate potential Z-DNA regions. Additionally, there is an About/Contact page with information on the tool’s algorithms and a help page explaining its features and functions.

**Figure 2:**
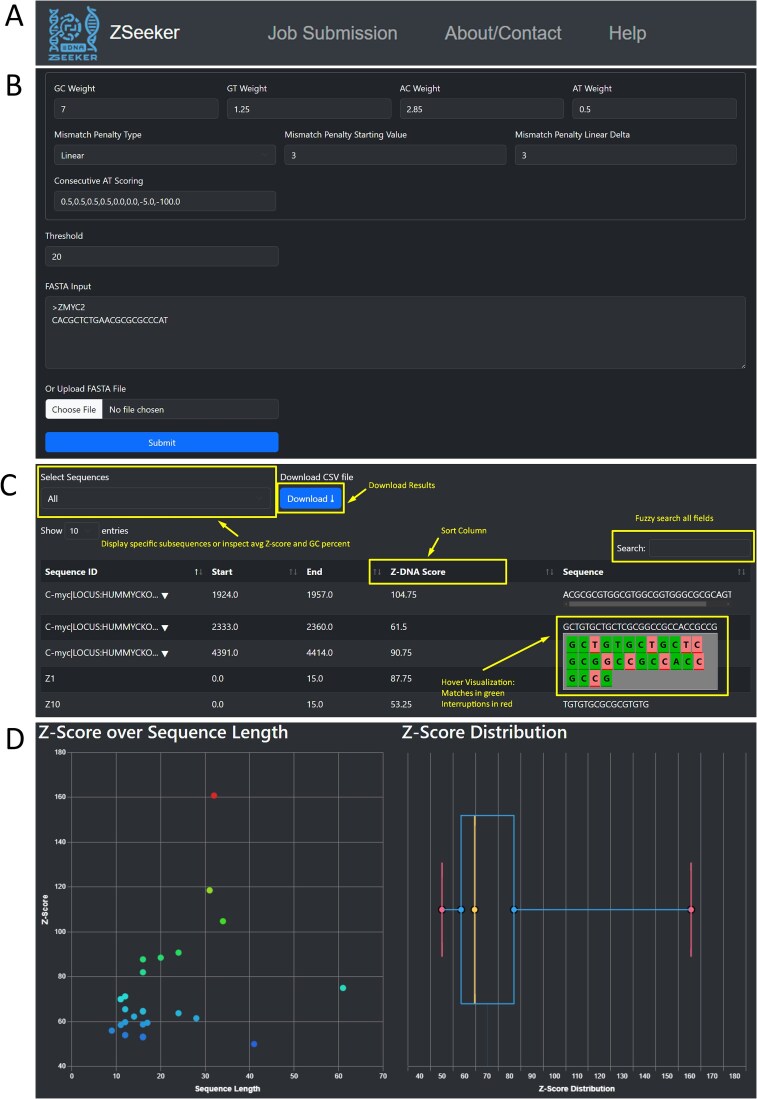
ZSeeker front page explaining the navigation to different pages and features. (A) Navigation bar for ZSeeker front pages. (B) The Job Submission page enables algorithm fine tuning. (C) The results section on the Job Submission page shows the Z-DNA sequences above the threshold score and provides users with the option filter, download, and sort features. (D) Visualizations are used to gain a quick overview of the results dataset.

The top navigation bar is composed of three tabs, the “Job Submission”, the “About/Contact”, and “Help” tabs, enabling navigation across the different parts of the ZSeeker website (Fig. 2A). The “Job Submission” page displays default parameters for performing Z-DNA detection, which can be adjusted by the user for their specific needs. The user can either paste a FASTA sequence in the input box or upload a FASTA file (Fig. 2B). After pressing the submit button, the file is processed, returning a set of outputs. These include a table and a CSV file with all potential Z-DNA sequences detected (Fig. 2C), and two visualizations, a scatter plot showing the Z-score and Z-DNA sequence length for all sequences detected, and a boxplot displaying the distribution of Z-scores for the input data (Fig. 2D).

**Figure 3:**
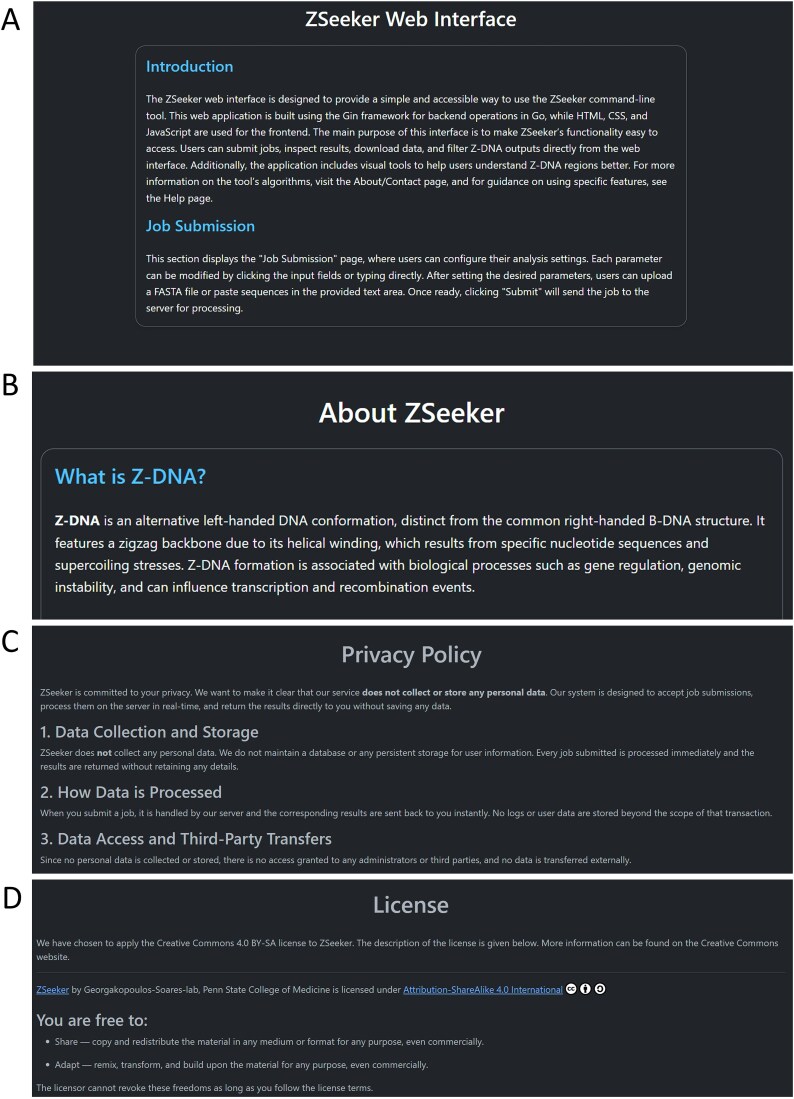
ZSeeker Help, About, and Privacy policy & License pages. (Α) Help page, (B) About page, (C) Privacy policy section, (D) License section.

The website contains a Help page, which provides information about Z-DNA to introduce the users to the ZSeeker program and guidance to the different features of the web app (Fig. 3A). An About/Contact page provides contact information for potential bug fixes and feature requests (Fig. 3B). A Privacy and License page is integrated that includes the website’s security, privacy policy (Fig. 3C,), and the license used, which is Creative Commons 4.0 BY-SA (Fig. 3D).

### Characterization of Z-DNA motifs in the human genome

We determined the total Z-DNA motif coverage across genomic subcompartments of interest in the human genome. In particular, we used genes, exons, silencers, enhancers, and coding regions, all extracted from RefSeq GFF annotations for CHM13v2-T2T *Homo sapiens* assembly with ID GCF_009914755.1 [38]. The additional 5′ UTR and 3′ UTR regions were annotated using Another Gff Analysis Toolkit (AGAT) [39]. The centromeric and pericentromeric regions were downloaded from https://github.com/marbl/CHM13. The total coverage of Z-DNA was calculated using bedtools coverage command, and we used Seaborn barplot with default parameters to calculate the average Z-DNA density across each genomic subcompartment, with error bars representing the standard error. Additionally, we isolated the genic regions from the RefSeq GFF annotations, using the command awk -F “\t” ‘$3 ~ gene && $0!~ /^#/’ {print $0}’ and keeping only protein-coding annotations. For each region, we expanded the TSSs and TESs in a symmetric 2000 base pair window both upstream and downstream. Subsequently, we used bedtools intersect -wo to map the Z-DNA motifs to each expanded region, calculated the relative distance from the TSS or TES and stored it in a 4001 numpy array, with the 2000th position representing the TSS or the TES for each gene. This process was repeated for each gene yielding a count vector with the total Z-DNA hits per relative position. The resulting count vector was normalized by dividing with the window average to estimate the enrichment of Z-DNA for each position. We obtained coordinates for stochastic and core origins from GSE128477 [40], originally aligned to the GRCh38 reference genome, and subsequently performed a liftOver to the CHM13v2-T2T human genome assembly. We quantified the enrichment of Z-DNA motifs relative to the replication origins using the same methodology described for TSSs and TESs. Each replication origin was defined by the midpoint between its start and end coordinates, around which we established a 4-kb analysis window. We then counted the occurrences of Z-DNA motifs at varying distances relative to the replication origins. Confidence intervals were estimated by performing Monte Carlo simulations with replacement (*n* = 1000) and determining the 2.5th and 97.5th percentiles from these simulations. Finally, to quantify enrichment, we normalized the observed Z-DNA counts by dividing by the average number of Z-DNA occurrences within the entire window and generated the mean profiles.

## Results

### The accuracy and efficiency of ZSeeker compared to other available programs

Among the various Z-DNA prediction algorithms available, Z-Hunt and its improved version, Z-Hunt II, are notable for their foundation in thermodynamic parameters, and have become widely recognized and accepted tools in the field. We tested the Z-Hunt II implementation currently provided by the Pui Shing Ho Lab (https://github.com/Ho-Lab-Colostate/zhunt) [19]. As the online version of this tool (http://zhunt.bmb.colostate.edu/) was unavailable at the time of writing of this paper (early 2025), we used the offline version provided on GitHub. However, even with modern computational resources, this 40-year-old algorithm is extremely slow, with suboptimal architecture and lack of documentation, and it is cumbersome for the users. For example, we have encountered several limitations of the tool. Firstly, the algorithm has a time complexity of O(2^n^) due to its exhaustive search through all possible anti-syn conformations for a DNA sequence of length n dinucleotides. For instance, with a window size of 20 dinucleotides, the program must evaluate ${2}^{20}$ (over 1 million) possible conformations for just one window, leading to extremely long computation times. This exponential growth makes the tool challenging to use for even medium-sized inputs unless the parameters minimum and maximum window sizes are set to very small values. Secondly, the original tool does not provide sequence coordinates within the larger DNA context and only includes the anti-syn structure with the lowest energy. This limitation reduces the practical utility of the results for biological applications where positional information is crucial. Additionally, the scores generated by the program, delta linking number, slope, and probability, are not clearly explained. There is insufficient documentation detailing what each score represents or which one should be considered indicative of Z-DNA propensity. Furthermore, there is no (suggested) cutoff in the output results to indicate if a sequence is considered as a Z-DNA motif. The program includes all sequences in the output regardless of their scores, making it difficult to distinguish between sequences with a high potential to form Z-DNA and those with low potential. This absence of a threshold complicates the analysis and makes it challenging to focus on the most relevant sequences.

Additionally, we encountered limitations regarding sequence lengths. Due to the dinucleotide basis of the Z-Hunt II algorithm, it cannot directly score sequences of odd length as a whole. For example, when analyzing the 17-nucleotide sequence GCGCGTGCATATGCGTG, Z-Hunt II cannot evaluate the entire sequence because it requires sequences composed of an even number of nucleotides to form complete dinucleotides. This means that the last nucleotide in an odd-length sequence cannot be incorporated into the analysis, potentially missing critical information. As a result, sequences such as GCGCGTGCATATGCGTG cannot be fully assessed by Z-Hunt II, limiting its applicability when dealing with sequences of varying lengths commonly found in genomic data. To demonstrate several of these challenges with Z-Hunt II, including its computational inefficiency, incomplete output, and lack of clear documentation, we used 20 Z-DNA-forming sequences that obtained Z-scores ranging from 31.7 to 1750 in the original publication [41] for this program, non-B_gfa and our novel algorithm, and the results are summarized in Table 2.

**Table 2:** Comparing the Z-DNA scores from ZSeeker, Z-Hunt II, Non-B DB and Z-DNABERT

Sequence	Zseeker Score (Rank)	Z-Hunt Score (Rank)	Non-B DB score (Rank)	Z-BERT score (Rank)
TGCGTGCGCGCGCGCG	87 (1)	1750.0 (1)	x^a^	0.732 (3)
GCGCCCGCGCGCGCGC	82 (2)	733.0 (2)	x	0.735 (2)
GCGCGCGCGCGT	71 (3)	303.0 (3)	x	0.208 (16)
CGCGCGCGCGC	70 (4)	148.0 (5)	125 (1)	0.281 (15)
GCGCGCGCGCG	70 (5)	129.0 (7)	x	0.205 (17)
GCGCGTGCGCGC	65 (6)	199.0 (4)	x	0.614 (6)
GCGCGCCCGTACGCGC	64 (7)	59.0 (12)	62 (3)	0.660 (4)
GCACGCACACGCGCGT	64 (8)	132.0 (6)	x	0.579 (9)
CGCACGCGCACGCA	62 (9)	103.0 (8)	x	0.554 (11)
CGCGCGCGCACA	59 (10)	75.0 (9)	x	0.442 (12)
TGTGCGCGCGCACATG	58 (11)	71.2 (11)	x	0.775 (1)
GCGCGCACGCG	58 (12)	39.4 (15)	103 (2)	0.440 (13)
CGCGCGCGC	56 (13)	37.9 (16)	x	0.283 (14)
GCGCACGCACGC	54 (14)	48.8 (13)	x	0.129 (19)
GCGCGCGCCCGC	54 (15)	37.9 (17)	x	0.185 (18)
TGTGTGCGCGCGTGTG	53 (16)	71.2 (10)	x	0.612 (7)
CGCGCACGCACACATG	53 (17)	46.4 (14)	x	0.570 (10)
GTGCGTGCCCGCGCGT	53 (18)	35.0 (19)	43 (4)	0.608 (8)
CACGCGCACGTGC	49 (19)	37.1 (18)	x	0.658 (5)
CGTGCGTGTGTGTGCG	47 (20)	31.7 (20)	x	0.121 (20)

^a^The sequence is not scored in Non-B DB and is not recognized as a Z-DNA-forming sequence.

The non-B DNA Motif Search Tool (nBMST) in the NCI non-B DB Genomic Database (https://nonb-abcc.ncifcrf.gov/apps/site/resources) is a more user-friendly tool than Z-Hunt II and is also very well accepted. Hence, we compared the search results from these two Z-DNA searching/prediction tools to the report from our ZSeeker program. Because Z-Hunt II (https://github.com/Ho-Lab-Colostate/zhunt) provides its output in an anti-syn structural form, we developed a modified version that logs instead the nucleotide sequences in the output to be able to compare with ZSeeker’s results.

Despite their fundamentally different approaches, Z-Hunt II and ZSeeker ranked the 20 sequences quite similarly, as shown in Table 2. The most obvious but still minor differences are the sequences ranked #7 and #16 by ZSeeker were ranked #12 and #10 by Z-Hunt II, respectively. In contrast, the nBMST failed to identify most (16/20) of the Z-DNA motifs that were reported in both ZSeeker and Z-Hunt II, including those having the highest scores. The deep learning Z-DNA prediction program, Z-DNABERT [42], scored the GC(5)GT sequence and the CG(5)C sequence, which were ranked #3 and #4 in ZSeeker and #3 and #5 in Z-Hunt II, among the lowest at #16 and #15 among 20 total sequences; and ranked the sequence CACGCGCACGTGC #5, which contains two CA pyrimidine-dinucleotides that interrupt the alternative purine-pyrimidine pattern and was scored low and ranked at #19 and #18 in Z-Seeker and Z-Hunt II, respectively. Thus, it appears that the nBMST lacks accuracy in predicting potential Z-DNA-forming sequences.

The current state of the art for predicting Z-DNA using machine learning are DeepZ [23] and Z-DNABERT [42]. DeepZ utilizes convolutional and recurrent neural networks along with DNA sequences and omics data to annotate Z-DNA regions. Z-DNABERT is a transformer-based model that uses DNA data to predict Z-DNA formation. Since ZSeeker in its core does not utilize omics data we decided for fairness to compare ZSeeker and Z-DNABERT only while omitting DeepZ from the comparison. To compare ZSeeker with Z-DNABERT the threshold derived from the transformer’s output layer was used to attribute a score to each of the Z-DNA sequences present at the comparison table. As a general observation, Z-DNABERT seemed to find the same two top ranking sequences with ZSeeker and Z-Hunt, but in general it follows a completely different approach in Z-DNA prediction based on context windows that yield very different results. Whereas all sequences present in the comparison table result in a threshold greater than 0.1, Z-DNABERT seems to annotate only regions and the ordering of most does not coincide with the results from Z-Hunt or ZSeeker. We believe that this is a result of the transformer architecture. Z-DNABERT was trained to find Z-DNA regions in a larger genomic context rather than assigning a large score to small but highly probable Z-DNA-forming sequences.

To further compare the accuracies of ZSeeker and Z-Hunt II in predicting Z-DNA-forming sequences, we tested sequences that have been confirmed to form Z-DNA structures in published literature using both programs. Visentin & Harley studied a sequence GCGCGTGACTATGCGTG in the mouse metallothionein I promoter [43] offering an ideal series of sample sequences to evaluate predictive accuracy. The native sequence has an alternating purine-pyrimidine element with an AC dinucleotide interruption in the middle. Reversing the interruption AC to CA created a consecutive alternating purine-pyrimidine pattern without changing the base content. Two-dimensional chloroquine gel analysis and diethyl pyrocarbonate sensitivity assays confirmed that a Z-DNA structure was formed on the mutant GCGCGTGCATATGCGTG sequence, while the native GCGCGTGACTATGCGTG was not able to adopt a Z-DNA structure unless a strong torsional stress was applied, while the CG(n) repeats and the GACGCGGGGCGCGTGCATATGCGTGG sequence formed Z-DNA structures even more readily than the mutant sequence [43]. Thus, these well-characterized sequences provide an ideal series of potential Z-DNA-forming sequences for validating the accuracies of the Z-DNA structure prediction models. ZSeeker scored the native GCGCGTGACTATGCGTG sequence 45.5, just below the cut-off score of 50; the mutant GCGCGTGCATATGCGTG sequence was scored 59.5 and was recognized as a Z-DNA-forming sequence; and the GACGCGGGGCGCGTGCATATGCGTGG sequence was scored 63.75 (Table 3). Thus, the predicting results matched the experimental results. By design, Z-Hunt II analyzes the DNA by dinucleotides and is not able to process any odd length sequences. As a result, the last base pair of the native and mutant sequences were omitted in this test. Interestingly, the truncated 16-bp native sequence that contains the AC interruption was scored highest (23); the 16-bp mutant sequence that has a consecutive alternating purine-pyrimidine pattern was scored 20, which is the same as the model sequence that was experimentally shown to be a much stronger Z-DNA-forming sequence. In contrast, the Z-DNABERT program failed to recognize any of the sequences as good Z-DNA-forming sequences and gave them very low scores. Notably, the native sequence was also scored higher than the mutant sequence. Thus, among these three models, ZSeeker provided the best prediction relative to the experimental results (Table 3).

**Table 3:** Comparing the Z-DNA scores from ZSeeker, Z-Hunt II, and Z-DNABERT

I.D.	Sequence	Zseeker score	Z-Hunt II score	Z-DNABERT score
Native	GCGCGTGACTATGCGT(G)^a^	45.5	23.28	0.002
Mutant	GCGCGTGCATATGCGT(G)^a^	59.5	19.95	0.001
Model	ACGCGGGGCGCGTGCATATGCGTG	63.8	19.97	0.511

^a^The last nucleotide (G) was disregarded for the Z-Hunt II calculation, as the tool only works on even-length sequences.

### Confirmation of parameters by sensitivity analysis

The sensitivity analysis revealed that the optimal performance of ZSeeker was achieved with a GC weight of 7, and GT and AC weights of 1.25, which were determined to be optimal as they consistently classified all test sequences in accordance with experimental expectations. Both 1.25 and 1 for GT and AC weights demonstrated similar counts; however, the selection of 1.25 was favored due to its better alignment with previously published Z-DNA formation experiments. A subsequent evaluation using a Python-based plotting script confirmed that the parameter combination of GC = 7, GT = 1.25, AC = 1.25, and AT = 0.5 reliably distinguished Z-DNA-forming sequences (scoring greater than 50) from nonforming ones (scoring less than 50). These findings support the robustness of the chosen parameter set and provide a flexible framework for further adjustments to account for additional variables, such as divalent cation concentrations and pH, that may influence Z-DNA stability. The results of the sensitivity analysis can be found in Supplementary Table S2.

### Genomic distribution of Z-DNA motifs in the human genome

**Figure 4:**
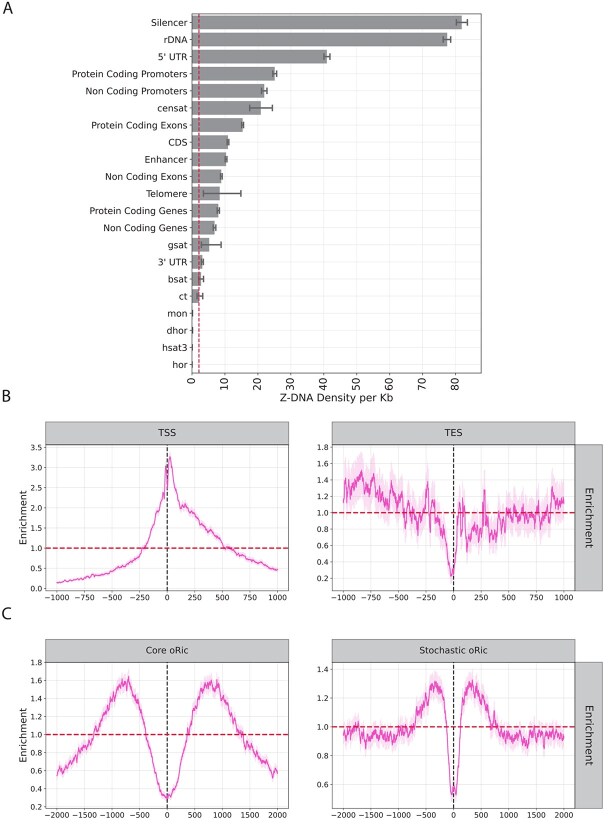
Characterization of Z-DNA motifs in human genomic subcompartments. (A) Z-DNA motif density across genomic sub-compartments. (B) Distribution of Z-DNA motifs relative to TSSs and TESs. (C) Distribution of Z-DNA motifs relative to core and stochastic oRic.

We examined the density of Z-DNA motifs in different regions of the human genome, including those relative to functional genomic elements. We identified 226 001 occurrences of Z-DNA motifs across the human genome, spanning a total of 6,597,193 base pairs, equivalent to an average density of 2.1 base pairs per kilobase. We first investigated which genomic compartments of the human genome contain the highest density of Z-DNA motifs. We report that silencers and ribosomal DNA array loci have the highest density, whereas centromeric repeats are the most depleted (Fig. 4A). To investigate the distribution of Z-DNA motifs in relation to TSSs and TESs across the human genome, we defined 500 bp analysis windows centered on these genomic sites. Within these regions, Z-DNA motif occurrences were quantified at each nucleotide position relative to the center. The results revealed significant enrichment of Z-DNA motifs near TSSs, with peak 3.3-fold enrichment observed at 25 bp downstream of TSSs (Fig. 4B). The distribution showed a clear symmetrical decline in Z-DNA motif frequency moving away from the center, reaching a minimum at 25 bp upstream of TESs, with background levels ~200 bp upstream and downstream. In contrast, at the TESs, we did not find enrichment of Z-DNA motifs (Fig. 4C). This is consistent with previous reports of the enrichment and roles of Z-DNA relative to TSSs in transcription initiation [9, 44, 45]. Additionally, we analyzed core and stochastic replication origins as previously detected by Akerman et al [40]. We observe that Z-DNA motifs are enriched at ~700 bp from either side of the core origins of replication (oRic), and 350 bp for stochastic oRic, exhibiting a maximum enrichment of 1.65-fold and 1.3-fold for core and stochastic replication origins, respectively. We conclude that Z-DNA motifs are highly enriched in silencers, ribosomal DNA arrays and 5′UTRs, are positioned in the vicinity of TSSs and are enriched near, but do not overlap with replication origins.

## Discussion

Accumulating evidence suggests that Z-DNA structures are involved in several biological and pathological processes and have roles in disease development and genetic evolution. The field is currently in urgent need of a new tool to analyze DNA sequences and predict the potential Z-DNA-forming motifs accurately and efficiently, from either a single sequence typed-in or pasted-in, or a large number of sequences for high-throughput analysis.

The development of ZSeeker represents a significant advance in the identification of sequences that have the potential to adopt Z-DNA structures. ZSeeker corroborates previous thermodynamic, biochemical, and cellular-based experimental data, and allows for more nuanced recognition of Z-DNA motifs across complex sequences. Compared with the Z-Hunt II, which laid foundational work in identifying potential Z-DNA regions, ZSeeker not only addressed significant gaps in computational efficiency, but also improved the accuracy by incorporating cellular-based experimental data to compensate for the limitations of data from 2-D gel analyses. In addition, ZSeeker is able to analyze a long DNA sequence and recognize the impact of sequence imperfections and the overlap and/or the interference from adjacent Z-DNA motifs in a long segment.

The formation of Z-DNA is a dynamic and complex process and is impacted by many factors that are not currently possible to include in traditional programs, including ZSeeker. Deep-learning models are based on large datasets that include multiple factors and features into the training, and thus may provide a more thorough approach. However, we found that the recent DeepZ program [23], which combines Z-DNA ChIP-seq data together with epigenetic codes, transcription factor and RNA polymerase binding sites, and open chromosome maps to search for Z-DNA-forming sequences, failed to recognize many obvious or experimentally determined Z-DNA-forming sequences in our test (Tables 2 and 3). A possible reason for the low accuracy is that the datasets used for training have biases based on the particular experimental conditions. For example, Z-DNA binding proteins interacting with the exposed Z-DNA in accessible regions, and the binding status of transcription factors or polymerases at the time of and under the specific conditions of the experiments. In addition, the datasets used for training were collected from different sources under different conditions, further reducing the power of the deep-learning programs. Therefore, without large numbers of high-quality unbiased datasets, the deep-learning programs are not mature enough at this stage.

Although ZSeeker significantly improves upon existing tools for detecting Z-DNA-forming sequences, certain limitations remain. The algorithm primarily relies on sequence composition and thermodynamic parameters informed by available experimental data, which may not fully capture the dynamic chromatin environment and structural variations occurring *in vivo*. Moreover, because prediction accuracy can vary based on user-defined parameters, careful adjustment of the default parameters is recommended to better model specific conditions. For example, if conditions are more favorable for Z-DNA formation (e.g. with magnesium ions or methylation), the user can change the threshold of Z-DNA detection accordingly. Finally, as a computational prediction tool, ZSeeker does not directly confirm Z-DNA formation experimentally, thus requiring further experimental validation.

Examination of Z-DNA-forming sequences with ZSeeker can be incorporated in studies related to genetic instability, genome organization and evolution, gene regulation, and human health and disease [2, 7, 36, 44, 46, 47]. ZSeeker features an intuitive web interface for seamless Z-DNA detection, offering outputs in the form of tables and visualizations that present Z-scores, penalties, and rank-ordered input sequences. Even though we recommend specific parameters to run ZSeeker as described herein, parameters can be altered enabling fine-tuning of the ZSeeker parameters for personalized searches. For larger datasets and programmatic use, a Python package is also available. By providing more accurate and adaptable Z-DNA detection, ZSeeker addresses a critical gap in current genomic analysis tools. We anticipate that our novel Z-DNA detection tool will encourage further research and exploration of Z-DNA within the scientific community.

**Key Points**

- Z-DNA plays important roles in many cellular processes yet current search tools have limitations that we have addressed by developing a novel Z-DNA search tool.
- ZSeeker is a novel computational tool developed for the accurate detection of potential Z-DNA-forming sequences based on experimental data.
- ZSeeker functions both as a standalone Python package and as an accessible user-friendly and high throughput web interface.

## Supplementary Material

Supplementary_Material_bbaf240

Supplementary_Table_1_Sensitivity_Test_Sequences_bbaf240

Supplementary_Table_2_Sensitivity_Analysis_Results_bbaf240

## Data Availability

ZSeeker is released as a Python package under the GPL license as a multi-platform application and is available at: https://github.com/Georgakopoulos-Soares-lab/ZSeeker. A web-interface of ZSeeker is publicly available at: https://zseeker.netlify.app/.

## Code availability

The ZSeeker package and its Python bindings are released under GPL license as a multi-platform application and are available at: https://github.com/Georgakopoulos-Soares-lab/ZSeeker and we also provide a dockerized version at https://github.com/Georgakopoulos-Soares-lab/ZSeeker_docker as well as our sensitivity analysis at https://github.com/Georgakopoulos-Soares-lab/zseeker_sensitivity_analysis. A stable version of ZSeeker is also found at: DOI: 10.5281/zenodo.15059513.
