# Supplementary material for: ZSeeker: an optimized algorithm for Z-DNA detection in genomic sequences
Source: Brief Bioinform. 2025 May 30;26(3):bbaf240. doi: 10.1093/bib/bbaf240 (PMC12123511; doi:10.1093/bib/bbaf240)
Supplement: Supplementary_Material_bbaf240 [file supplementary_material_bbaf240.docx]

**Supplementary Material for:**

ZSeeker: An optimized algorithm for Z-DNA detection in genomic sequences

Guliang Wang^1,*^, Ioannis Mouratidis^2,3,*^, Kimonas Provatas^3^, Nikol Chantzi^2,3^, Michail Patsakis^3^, Ilias Georgakopoulos-Soares^2,3,+^, Karen Vasquez^1,+^

^1^ Division of Pharmacology and Toxicology, College of Pharmacy, The University of Texas at Austin, Dell Pediatric Research Institute, Austin, TX, USA

^2^ Institute for Personalized Medicine, Department of Biochemistry and Molecular Biology, The Pennsylvania State University College of Medicine, Hershey, PA, USA.

^3^ Huck Institute of the Life Sciences, Pennsylvania State University, University Park, PA, USA

* Co-first authors

^+^ Corresponding authors: [izg5139@psu.edu](mailto:izg5139@psu.edu), [karen.vasquez@austin.utexas.edu](mailto:karen.vasquez@austin.utexas.edu)

**
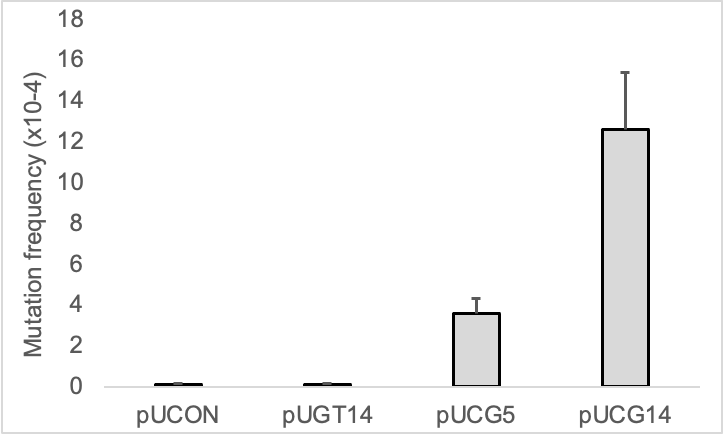
**

**Supplementary Figure 1: GT and CG repeat-induced mutation frequencies in DH5alpha cells.** GT14, CG5 and CG14 model sequences were cloned in a lacZ’ mutation reporter and the mutation frequencies were screened after the plasmids were replicated in DH5alpha cells for 16 hours. pUCON contains a random 28-bp sequence as a control. Error bars show the standard error of the mean from >3 independent repeats.


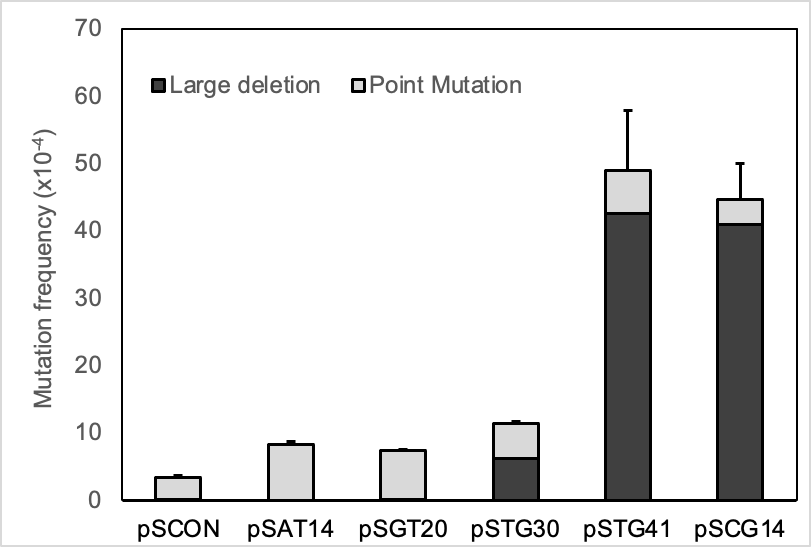


**Supplementary Figure 2: Repeat-induced mutation frequencies in mammalian COS-7 cells.** AT14, GT20, GT30, GT41 and CG14 model sequences were cloned in a supF mutation reporter and the mutation frequencies were screened after the mutation-reporter plasmids were transfected and replicated in COS-7 cells for 48 hours. pSCON contains a random 28-bp sequence as a control. >20 mutants were randomly picked and sequenced to determine the mutation spectra. Dark bars represent the frequencies of Z-DNA-induced large deletions resulting from DSBs, and light gray bars represent the frequencies of point mutations and small indels. Error bars show the standard error of the mean from >3 independent repeats. Results obtained for GT30 and GT41 were published as part of figure in [[32]](https://paperpile.com/c/TqvvCh/x8M3).

Also, please see attached:

**Supplementary Table 1 Sensitivity Test Sequences.xlsx**

**Supplementary Table 2 Sensitivity Analysis Results.xlsx**
